# Editorial: The NLRP3 inflammasome-mediated neuroinflammation and its related mitochondrial impairment in neurodegeneration

**DOI:** 10.3389/fnagi.2022.1118281

**Published:** 2023-01-11

**Authors:** Chao Deng, Xiang Cai, Kunlin Jin, Qing Wang

**Affiliations:** ^1^School of Medical, Indigenous and Health Sciences, University of Wollongong, Wollongong, NSW, Australia; ^2^Antipsychotic Research Laboratory, Illawarra Health and Medical Research Institute, Wollongong, NSW, Australia; ^3^Center of Depression, Beijing Institute of Brain Disorders, Advanced Innovation Center for Human Brain Protection, Collaborative Innovation Center for Brain Disorders, Capital Medical University, Beijing, China; ^4^Department of Pharmacology and Neuroscience, University of North Texas Health Science Center, Fort Worth, TX, United States; ^5^Department of Neurology, Zhujiang Hospital, Southern Medical University, Guangzhou, Guangdong, China

**Keywords:** neurodegeneration, NLRP3, neuroinflammation, mitochondrial, δ-opioid receptors, aging

Inflammasomes are multiprotein complexes of the innate immune system that play critical roles in activation of a variety of inflammatory responses. Many inflammasomes have been identified. Among of them, the nucleotide-binding oligomerization domain-like receptor pyrin domain-containing-3 (NLRP3) has been well characterized (Guo et al., 2015; Zheng et al., 2020). Recently, NLRP3 inflammasome caught neuroscientists' attention, as the NLRP3 inflammasome has been demonstrated to mediate neuroinflammation in various neurodegenerative diseases (Holbrook et al., 2021; Anderson et al., in press). The NLRP3 inflammasome is a cytosolic multiprotein complex composed of intracellular innate immune receptor NLRP3, junction protein ASC and protease caspase-1 (cysteine aspartate protease 1) (Guo et al., 2015). Activated NLRP3 inflammasomes can induce the maturation and secretion of pro-inflammatory factors such as IL-1β and IL-18 in microglia, thereby promoting the neuroinflammation associated with neurodegenerative diseases, including Alzheimer's disease (AD), Parkinson's disease (PD), amyotrophic lateral sclerosis (ALS), as well as ischemic stroke (Holbrook et al., 2021; Anderson et al., in press). Therefore, the NLRP3 inflammasome has progressively moved into the spotlight leading to the creation of a new research area on the pathogenesis of neurodegenerative diseases.

Previous studies have found that mitochondrial impairment is an important driving force leading to excessive activation of the NLRP3 inflammasome (Holbrook et al., 2021; Mishra et al., 2021). Recently, more studies have been devoted to better understanding NLRP3 inflammasome-mediated neuroinflammation and its relationship with mitochondrial damage in neurodegenerative diseases (Mishra et al., 2021). Despite these efforts, the biological mechanisms underlying NLRP3 inflammasome-mediated progression of neurodegenerative diseases, and how to maintain mitochondrial homeostasis to prevent excessive activation of NLRP3, remain unclear both *in vivo* and *in vitro*.

This Frontier Research Topic has brought together a group of leading experts in the field to address these critical issues. Three of the articles in this Research Topic explored the underlying mechanisms of the NLRP3 inflammasome and their potential in PD and AD therapeutics by targeting the NLRP3 inflammasome. In a perspective article, Su et al. discussed the key role of a-synuclein and Parkin, two PD-associated proteins, in NLRP3 activation and PD pathogenesis. They also discussed pre-clinical outcomes of several NLRP3 inflammasome inhibitors (such as MCC950, Kaempferol, and miRNA-7) in treating PD and other neurodegenerative diseases (Su et al.). Using an MPTP-induced mouse model and 6-OHDA induced SH-SY5Y cell model, Que et al. investigated the therapeutic potential of Dl-3-nbutylphthalide (NBP) for treating PD. Their findings showed that NBP could rescue dopaminergic neurons by reducing NLRP3 inflammasome activation and the aggregation of a-Syn, leading to amelioration of mitochondrial impairments (Que et al.). Since the NLRP3 inflammasome is activated by amyloid β (Aβ) and/or ATP *via* P2X7R in microglia, targeting P2X7R is a plausible approach to resolve neuroinflammation in AD patients. Islam et al. tested the effects of taurodeoxycholate (TDCA, a GPCR19 ligand) in inhibiting the activation of NLRP3 inflammasome and P2X7R expression. Their data provided important evidence that TDCA was also effective in decreasing Aβ plaques and preventing neuronal loss, along with improving memory dysfunction in the 5xFAD mouse model of AD (Islam et al.).

Two papers on this Issue investigated the neuroprotective roles of δ-opioid receptors (DOR) and DOR-mediated neuroinflammation signaling pathways (including NLRP3 inflammasome) in hypoxic/ischemic insults. Chen et al. compared the effects of DOR on the expression of miRNAs in hypoxic/ischemic-sensitive organs (such as brain, kidney, and heart) and hypoxic/ischemic-insensitive organs (such as liver and muscle), and their impact on inflammatory injury. On the other hand, Xu et al. reported that UFP-512, a specific DOR agonist, inhibited hypoxia-induced microglial M1 activation and inflammatory activity, while knocking-down or inhibiting DOR promoted microglial M1 and M2 activations.

Thioredoxin-interacting protein (TXNIP, an α-arrestin protein) plays a regulator role in inflammation and various neurodegenerative diseases (Tsubaki et al., 2020). Cheng and Wang introduced a novel approach to study the interactions of TXNIP and NLRP3 in three *in silico* models to evaluate the binding stability of the possible interaction of TXNIP/NLRP3. Their data suggest that the N-terminal of TXNIP is essential in allosteric regulation of NLRP3, even without directly binding to NLRP3, which provides an invaluable insight for future development of NLRP3 inflammasome inhibitors (Cheng and Wang).

In summary, studies published in this Research Topic provide an overview of the role of the NLRP3 inflammasome in the pathogenesis of PD, AD, hypoxia and ischemia. In particular, these articles investigated drugs that regulate NLRP3 inflammasome-related damage and potential therapies for these neurodegenerative diseases. Their findings provided important evidence to support inhibition of the NLRP3 inflammasome as a promising therapeutic target, including NLRP3 inflammasome inhibitors (such as MCC950 and NBP) for the treatment of PD, a GPCR19 ligand (e.g., TDCA) for the treatment of AD, and δ-opioid receptors for hypoxic/ischemic treatment (Chen et al.; Islam et al.; Que et al.; Su et al.; Xu et al.). Furthermore, the constructed models of TXNIP/NLRP3 interaction are also valuable for inhibitor development targeting the TXNIP/NLRP3 interaction during inflammasome activation (Cheng and Wang). We believe that this Frontier Research Topic will stimulate additional research by taking innovative approaches for further revealing the role of NLRP3 inflammasome in the pathogenesis of neurodegenerative diseases, and identifying new medications for the treatment of these disorders.

## Author contributions

CD prepared the initial draft of the manuscript. CD, XC, KJ, and QW revised the manuscript. All authors contributed to the article and approved the submitted version.
